# Robust License Plate Recognition in OCC-Based Vehicle Networks Using Image Reconstruction

**DOI:** 10.3390/s24206568

**Published:** 2024-10-12

**Authors:** Dingfa Zhang, Ziwei Liu, Weiye Zhu, Jie Zheng, Yimao Sun, Chen Chen, Yanbing Yang

**Affiliations:** 1 College of Computer Science, Sichuan University, Chengdu 610065, China; zhangdingfa@pku.edu.cn (D.Z.); ziweiliuofficial@outlook.com (Z.L.); waynechuofficial@gmail.com (W.Z.); yangyanbing@scu.edu.cn (Y.Y.); 2 CPU Design Center, Haiguang Integrated Circuit Design Co., Ltd., Chengdu 610095, China; buluo888200@163.com; 3 Institute for Industrial Internet Research, Sichuan University, Chengdu 610065, China; 4 School of Microelectronics and Communication Engineering, Chongqing University, Chongqing 400044, China; c.chen@cqu.edu.cn

**Keywords:** internet of vehicle (IoV), optical camera communication (OCC), license-plate recognition (LPR), vehicle to infrastructure (V2I)

## Abstract

With the help of traffic lights and street cameras, optical camera communication (OCC) can be adopted in Internet of Vehicles (IoV) applications to realize communication between vehicles and roadside units. However, the encoded light emitted by these OCC transmitters (LED infrastructures on the roadside and/or LED-based headlamps embedded in cars) will generate stripe patterns in image frames captured by existing license-plate recognition systems, which seriously degrades the accuracy of the recognition. To this end, we propose and experimentally demonstrate a method that can reduce the interference of OCC stripes in the image frames captured by the license-plate recognition system. We introduce an innovative pipeline with an end-to-end image reconstruction module. This module learns the distribution of images without OCC stripes and provides high-quality license-plate images for recognition in OCC conditions. In order to solve the problem of insufficient data, we model the OCC strips as multiplicative noise and propose a method to synthesize a pairwise dataset under OCC using the existing license-plate dataset. Moreover, we also build a prototype to simulate real scenes of the OCC-based vehicle networks and collect data in such scenes. Overall, the proposed method can achieve a recognition performance of 81.58% and 79.35% on the synthesized dataset and that captured from real scenes, respectively, which is improved by about 31.18% and 24.26%, respectively, compared with the conventional method.

## 1. Introduction

Deemed as an important technology to achieve Internet of Vehicles (IoV) communication, optical camera communication (OCC) has gathered much attention from both academic and industrial communities, thanks to its inherent superiority of availability [1,2,3,4]. The integration of OCC into automotive and intelligent transportation systems(ITS) has hence also been extensively researched [5,6,7,8]. Typically, OCC can be particularly built upon pervasive LED lighting infrastructures as transmitters and embedded cameras as receivers for establishing communication, where both LEDs and cameras are the default equipment on vehicles or roadside units presently [1]. Generally, the application scenarios for OCC in IoV can be categorized into two types, namely vehicle-to-vehicle (V2V) and vehicle-to-infrastructure (V2I). The former employs vehicle-mounted LED lamps and cameras as transceivers to transmit information between adjacent vehicles [6,7]. The latter uses LED lights and cameras as transmitters and receivers on vehicles or roadside units to exchange messages between vehicles and roadside units, such as traffic lights and monitors [1,8,9].

**Figure 1:**
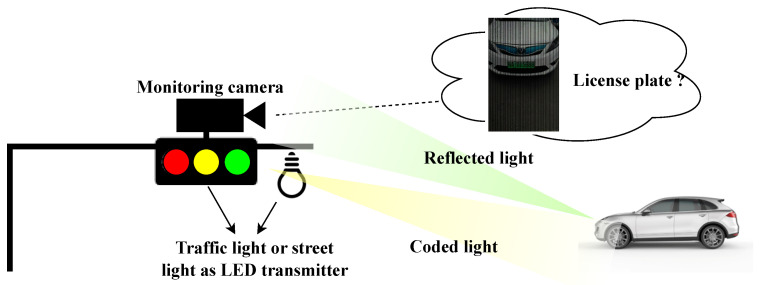
In applications of the IoV that adopt OCC, license-plate recognition cameras can interfere with coded light emitted from OCC devices; thus, the recognition performance is affected.

As shown in Figure 1, a typical V2I application scenario consists of a roadside unit (traffic light and camera) and vehicles. A camera is often fixed on the roadside to record images or videos for traffic monitoring. At the same time, it functions as an OCC receiver, receiving messages transmitted from vehicles. Meanwhile, to mitigate the rolling shutter effect in shooting moving targets, e.g., moving cars, the exposure time to less than 0.001 s [10], which is also a requirement of OCC to achieve relatively higher data rate [11]. However, in such a case, the captured images are mixed with the vehicle’s picture and OCC data carried by the bright and dark stripes. These stripes inevitably degrade the basic sensing functions of the monitoring camera, such as license-plate recognition. Therefore, a dual-camera scheme is proposed in [9] to realize simultaneous traffic sign recognition and real-time OCC using two cameras, where one is for conventional image recognition and the other for OCC. Obviously, it is impractical to employ two cameras in a real ITS or IoV application in our daily lives.

As for stripe noise elimination in image recognition, some researchers propose using filter-based methods to eliminate noise stripes in images. For example, Zhang et al. [12] construct a kind of adaptive frequency filter based on 2-D fast Fourier Transforms for image de-striping. Münch et al. [13] combines wavelet and Fourier analysis to eliminate the horizontal or vertical stripes in images. Lee et al. [14] use a method built upon a framework that includes denoising and rectification to obtain the high-quality license-plate image from the low-quality one. Moreover, a method is proposed to leverage optimization with the auxiliary tasks for multitask fitting and novel training losses. They view high-quality images as ground truths and obtain low-quality images through downsampling for training. Moreover, to eliminate the impact of noise and obtain high-quality images unaffected by car beams and streetlights, some works focus on enhancing nighttime license-plate images. They integrate the quotient image technique to address illumination variations and apply homomorphic filtering to remove noise, all within one framework [15]. Nevertheless, these methods either focus on removing noise of known distribution or manually reduce the quality of license-plate images for recovery. While current license-plate recognition systems are interfered with by OCC stripes, these stripes emerge as a non-temporal variation and belong to an uncertain distribution. Thus, current works are unable to cope with this challenge effectively.

To address this, we propose a simple and effective license-plate recognition (LPR) scheme. It includes an advanced image reconstruction (IR) module to restore local pixel details in images corrupted by OCC stripes. The image reconstruction module involves an end-to-end deep denoising network trained to learn the distribution of the images without OCC stripes. To address the issue of insufficient data in OCC-based vehicle networks, we model the OCC noise as multiplicative noise, and we also propose a method to synthesize a pairwise dataset under OCC using the existing license-plate dataset. Additionally, we introduce a noise factor to control the intensity of the noise, making the entire training process smoother. Meanwhile, we build a prototype and collect data to evaluate the performance in real scenes of the OCC-based vehicle networks. We conducted extensive experiments, and the results show that the proposed method significantly repairs the local details of the corrupted license plate. The reconstructed license-plate frames achieve an average recognition accuracy of about 80% under various experimental settings.

The rest of the paper is organized as follows. Section 2 presents the architecture of our license-plate recognition scheme and introduces each main module in this scheme, respectively. Section 3 explains the implementations, including a prototype and dataset building. Section 4 reports the performance of the proposed scheme in terms of detection accuracy and recognition accuracy and then discusses the experimental results. Finally, Section 5 concludes the whole paper.

## 2. LPR Scheme in the OCC-Enabled Vehicle Network

**Figure 2:**
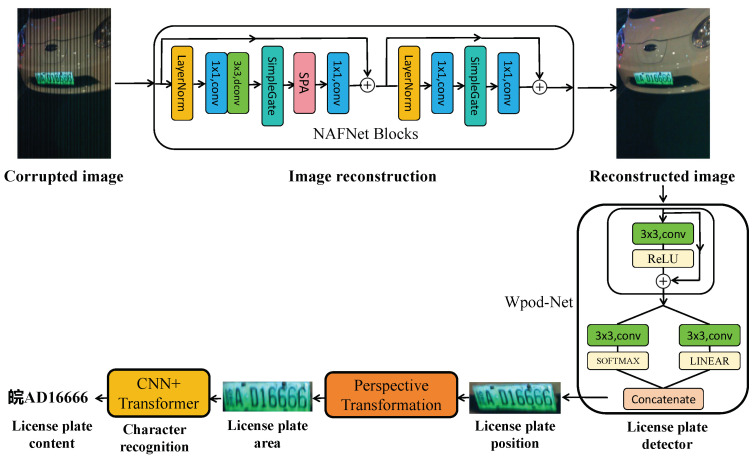
Diagram of proposed license-plate recognition scheme workflow in the vehicle networks.

Figure 2 briefly shows the proposed pipeline scheme architecture and modules for the vehicle networks enabled by OCC technology. The scheme is composed of three main components: the image reconstruction module, the license-plate detection module, and the license-plate character-recognition module. First, the license-plate frames mixed with vehicle and OCC stripes are captured by the LPR camera, and the image reconstruction module is used to generate clean frames without OCC interference. The clean license-plate frames are then fed into the detection module to build affine matrixes that transform the certain square area into the warped license-plate region [16]. Once the region of the license plate is extracted, a perspective transformation is applied to rectify the distorted license plate from oblique views to a correct perspective. Finally, the license-plate character-recognition module extracts the text information from the rectified license-plate. These modules constitute the proposed LPR scheme, which is suitable for OCC-based vehicle networks. The following sections will provide a detailed explanation of each module.

### 2.1. Image Reconstruction Module for Corrupted License Plate

In the OCC-based vehicle networks, the captured license-plate frames are mixed with the OCC stripes. Here, let $x_{\mathrm{original}}$ be the original frame without OCC stripes, and y means the OCC frame. To mitigate the neglective effect of OCC strips on LPR, we devise an image reconstruction module. In particular, we leverage the NAFNet [17], which is a U-shaped network structure architecture with skip connections. The NAFNet adopts convolution networks in stacked blocks considering the simplicity of depthwise convolution, and also replaces the ReLU activation function [18] with designed SimpleGate, which directly divides the feature map into two parts in the channel dimension and multiply them:

(1) SimpleGate(X,Y)=X⊙Y,

where X and Y are feature maps divided in channel dimension of the same size and ⊙ means the element-wise multiplication, and then use the simplified channel attention (SCA) in order to further simplify the whole structure and its complexity in the calculation:

(2) $$\mathrm{SCA}\left(X\right)=X*W{\mathrm{pool}}_{c}\left(X\right)$$

where ${\mathrm{pool}}_{c}$ indicates the global average pooling operation which aggregates the spatial information into channels, and W indicates the projection layer. Inspired by SCA, we introduced a module named simplified spatial attention (SPA), which has a similar expression:

(3) $$\mathrm{SPA}\left(X\right)=X*W{\mathrm{pool}}_{s}\left(X\right)$$

where ${\mathrm{pool}}_{s}$ indicates the global average pooling operation which aggregates the channel information into spatial.

With the preparations above, the NAFNet receives y as input and gives an estimation of the clean frame written as $x_{\mathrm{recon}}$, and we use the Charbonnier penalty function [19,20] as the loss function since it obtains better performance and requires fewer iterations compared with the $l_{2}$ loss function [20]. Here, it can be implemented as:

(4) $$\mathrm{loss}\left(x_{\mathrm{recon}},x_{\mathrm{original}}\right)=\sqrt{∥x_{\mathrm{recon}}-x_{\mathrm{original}}{∥}^{2}+\epsilon^{2}},$$

where ϵ is a constant that maintains the gradient of loss function presence and smooth convergence. The module is trained on patches set as 256×256 cropped from images randomly and is tested on full-resolution images. Notably, this operation brings performance degradation and patch boundary artifacts if we choose to test on patches. To solve the problem, we adopt the Test-time Local Converter (TLC) to convert the global operations in the network to local operations only during inference [21].

### 2.2. Reconstructed License-Plate Detection Module

License plates are usually captured as irregular quadrilaterals during the recognition process. In order to appropriately capture the shape of the license plate, the license-plate detection module adopts the Wpod-Net [16], which accepts the vehicle images as input and results in an 8-channel feature map. These parameters in the 8-channels indicate whether there exists an object to be detected at each point in the feature map and coefficients for affine transformations.

We first consider the vehicle images with $p_{i}={\left[x_{i},y_{i}\right]}^{T}$, for i=1,2,3,4 as the four corners of the area of a license plate, and let $q_{1}={\left[-0.5,-0.5\right]}^{T},q_{2}={\left[0.5,-0.5\right]}^{T},q_{3}={\left[0.5,0.5\right]}^{T},q_{4}={\left[-0.5,0.5\right]}^{T}$ be the corresponding vertices of a canonical unit square centered at the origin. For each point, (m,n) in the M×N feature map, Wpod-Net gives 8 values, and the first two values indicate the confidence concerning the existence of objects, and the last six values are then used to build the affine transformation $T_{mn}$:

(5) $$T_{mn}\left(q_{i}\right)=\left[\begin{array}{cc} max(v_{3},0) & v_{4}\\ v_{5} & max(v_{6},0) \end{array}\right]+\left[\begin{array}{c} v_{7}\\ v_{8} \end{array}\right],$$

where the $v_{i}$ represent the sorted 8 values, and then the $p_{i}$ is re-centered according to the point (m,n) in the feature map:

(6) $$A_{mn}\left(p_{i}\right)=\frac{1}{\alpha }\left(\frac{1}{N_{s}}p-\left[\begin{array}{c} n\\ m \end{array}\right]\right),$$

where α is a scaling constant that represents the side of the fictional square and $N_{s}$ represents the total stride of the downsampling process of the network; thus, the whole loss function of Wpod-Net can be expressed as:

(7) $$\begin{array}{cc} loss(p,q,v) & =\sum_{m=1}^{M}\sum_{n=1}^{N}\left[I_{\mathrm{obj}}f_{\mathrm{affine}}\left(m,n\right)+f_{\mathrm{probs}}\left(m,n\right)\right]\\ \; & =\sum_{m=1}^{M}\sum_{n=1}^{N}[I_{\mathrm{obj}}\sum_{i=1}^{4}{∥T_{mn}\left(q_{i}\right)-A_{mn}\left(p_{i}\right)∥}_{2}\\ \; & -I_{\mathrm{obj}}log\left(v_{1}\right)+\left(I_{\mathrm{obj}}-1\right)log\left(v_{2}\right)], \end{array}$$

the loss function first makes the warped unit square and normalized annotated points close enough and then measures the confidence concerning the object’s appearance at a certain point with the cross-entropy function.

Normally, the object to be detected is considered at the point of (m,n) if its rectangular bounding box presents an IoU larger than the given threshold, and $I_{\mathrm{obj}}$ in the loss function is a state function which returns 1 if the object exists or returns 0 otherwise. After obtaining the inference of the location of the license plate, we then adopt perspective transformation to rectify the possibly distorted license plate due to oblique views into a positive perspective, and this function was built with the help of OpenCV [22].

### 2.3. License-Plate Character-Recognition Module

After locating the license-plate area, character segmentation is used for obtaining license-plate characters. However, traditional text recognition algorithms such as fixed character spacing and connected component analysis easily lead to errors in segmentation may affect the recognition process, thus degrading the accuracy of the entire module. Here, we leverage [23] that recognizes characters without segmentation processes, which effectively avoids these errors considering the complex distribution of noise in the OCC-based vehicle networks. The segmentation-free methods transform the license-plate recognition problem into a character sequence labeling problem utilizing the global information of the input image. Inspired by this work, we adopted a CNN [24]+Transformer [25]-based character-recognition module, where the CNN extracts the reconstructed image features and the transformer is used to decode these features into text.

## 3. Prototype Implementation and Dataset

**Figure 3:**
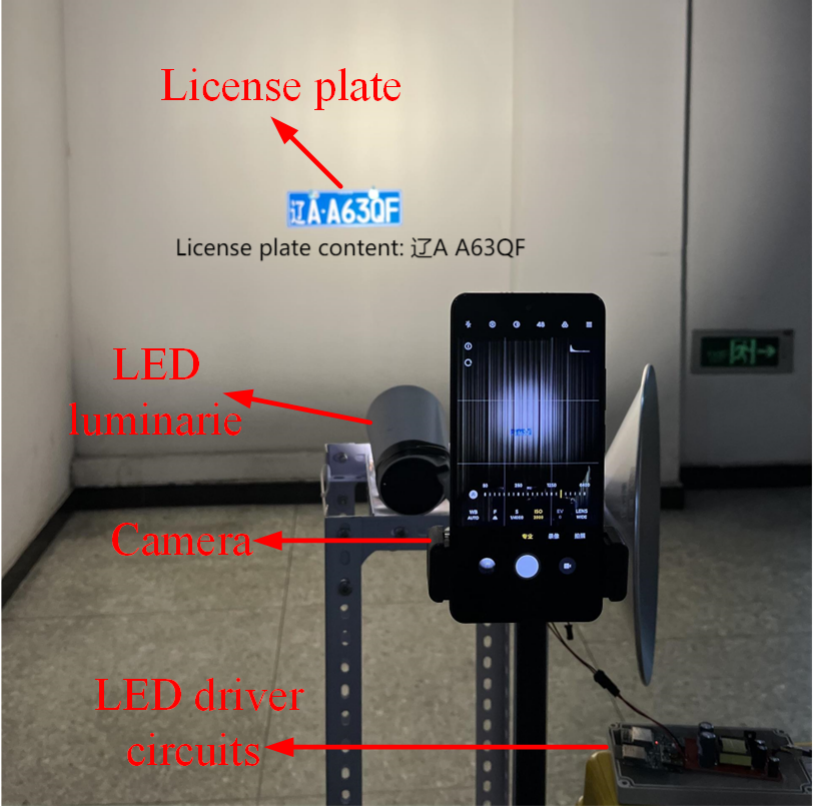
We build a prototype that consists of a 30 W LED to simulate the LED infrastructures on the roadside in the OCC-based vehicle network and a Redmi K40 to simulate the LPR camera. We use this prototype to collect frames that are then used to build our dataset.

To verify our idea, we build a prototype as shown in Figure 3 to generate a license-plated dataset with OCC strips. Specifically, to simulate the LED infrastructures on the roadside in the OCC-based vehicle network systems, we use a 30 W LED luminaire as the OCC system transmitter, which is controlled by an ARM Cortex-M4 GD32F330G8U6 microcontroller (GigaDevice Semiconductor Inc, Beijing, China) [26] to generate the digitally controlled signal for light modulation of On-Off keying (OOK). On the receiver side, we employ an embedded camera to capture modulated light reflected from the license plate with different parameter settings to mimic the traffic camera on the road.

**Figure 4:**
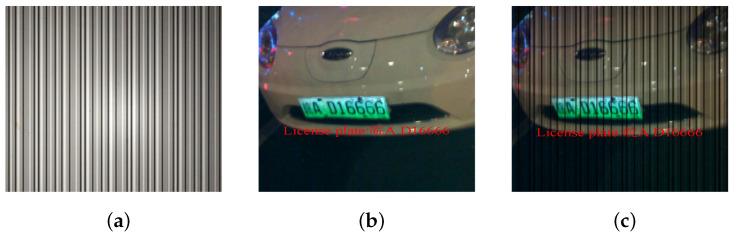
Synthesize a dataset of OCC noise and original image. (**a**) OCC noise. (**b**) Original image. (**c**) Synthesized image.

To train the image reconstruction module, we need a pairwise dataset that contains the OCC noised images and clean images. As there is no available public dataset for license-plate recognition under the interference of OCC, and collecting a massive real-scene dataset is also excessively labor-intensive, we hence design a method that adopts the existing license-plate recognition dataset (such as the CCPD) to build a new dataset for our work as shown in Figure 4. In particular, we consider the OCC stripes in the images as multiplicative noise. Mathematically, let $x_{\mathrm{original}}$ be the clean image from the license-plate dataset without the inference of OCC and $x_{\mathrm{occ}}$ means the stripe pattern noise images, then we build the corrupted image y as:

(8) $$y=x_{\mathrm{original}}⊙x_{\mathrm{occ}},$$

where ⊙ means the element-wise multiplication.

**Figure 5:**
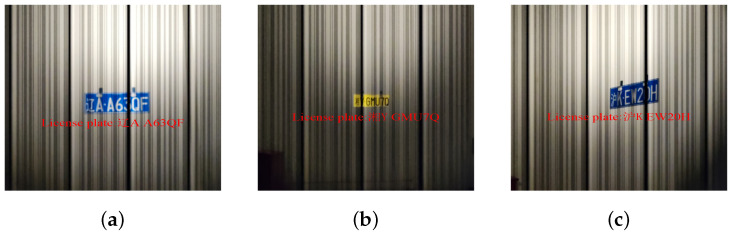
Examples of the captured dataset from real OCC-based vehicle network scene. (**a**) Distance 2 m, angle $0^{\circ }$. (**b**) Distance 4 m, angle $0^{\circ }$. (**c**) Distance 3 m, angle $60^{\circ }$.

To be more general, we introduce the noise factor α in the range of 0 to 1 to control the proportion of noise components in the synthetic image, and then the corrupted image y can be written as:

(9) $$\frac{1}{2}\ln y=\alpha \ln x_{\mathrm{original}}⊙\left(1-\alpha \right)\ln x_{\mathrm{occ}},$$

and normally when α equals 0.5, the above equation is equivalent to Equation (8). Inspired by the human learning process, we gradually increase the noise factor α from 0.4 to 0.5 during training. This shifts the process from easy to difficult, allowing the model to better learn the representation of noise in the images. The stripe-structured noise can distort the brightness of the original frames in a multiplicative form, depending on the differences in the distribution of gray values. This distortion creates visual effects where background objects are interfered with by OCC light. To address this, the captured frames are used as multiplicative noise to build a pairwise dataset with the CCPD dataset. We remove some images of poor quality and randomly choose 1000 images from CCPD-db to test and use the rest to build the pairwise dataset and train the image reconstruction module. Moreover, to evaluate the performance of the proposed scheme in real scenes of the OCC-based vehicle networks, we also adopt the prototype to make a dataset in these scenes as shown in Figure 5. Considering the variety of distances and angles between the vehicle and LPR system in real scenes, we mainly explore the impact of the two parameters on system performance. For each combination of the two parameters, we collect 200 images, so the dataset ends up containing over 1600 real-scene images.

As for the whole recognition software settings, we adopt the NAFNet architecture with a width of 32 in the image reconstruction module, the number of stacked encoder blocks and decoder blocks are 2,2,4,8, and 2,2,2,2, respectively. The module is trained for 200 epochs upon patches size 256×256 randomly cropped from the original images using AdamW [27] optimizer with $\beta_{1}=0.9,\beta_{2}=0.9$, the initial learning rate is $1\times 10^{-3}$ and gradually reduces to $1\times 10^{-6}$ with the cosine annealing schedule [28]. To obtain a detection model with better generalization ability, we adopt ResNet-18 [29] as the feature extraction module of Wpod-Net. We first train the model upon the CCPD-base dataset [30] which has over 200,000 unique images with annotations for LPR, and then fine-tune on the CCPD-db dataset which is a subset of the whole CCPD dataset and contains over 10,000 images where the illuminations are dark or uneven. We also adopt random Gaussian noise for data augmentation to improve the robustness of the module. After locating the area of the license plate, we then adopt the license-plate character-recognition module to recognize the characters on the license plate. The license-plate character-recognition module consists of a ResNet structure and two stacked transformer blocks with hidden dimensions of 512 and is trained for over 100 epochs. Since it is unrealistic to collect substantial labeled license-plate images for the license-plate character-recognition module, we randomly generate simulated license-plate images, which are annotated during the generation process.

## 4. Experimental Results and Discussions

**Figure 6:**
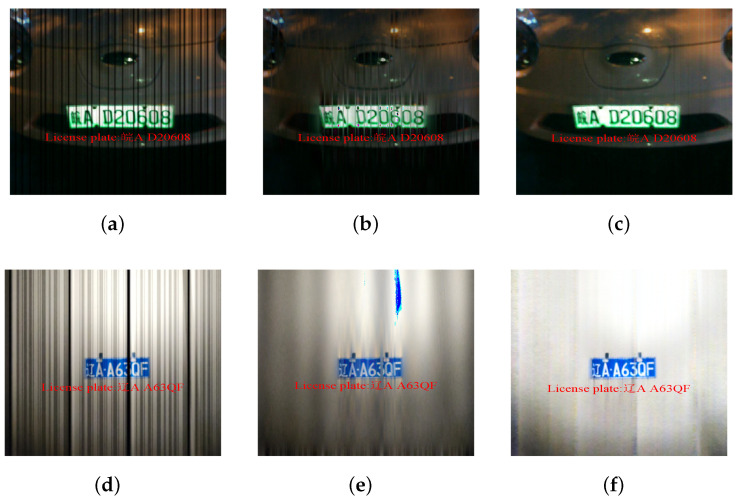
A visualization comparison on synthesized image and real-scene image. (**a**) Synthesized OCC image. (**b**) Result of band-pass filter on the synthesized dataset. (**c**) Result of IR module on the synthesized dataset. (**d**) Real-scene OCC image. (**e**) Result of band-pass filter in real scenes. (**f**) Result of IR module in real scenes.

In this section, we extensively evaluate the performance of the proposed LPR scheme on the synthesized dataset and in real scenes of the OCC-based vehicle networks. The baseline is defined as directly detecting and recognizing the license plate mixed with OCC interference. We also adopt the band-pass filter to eliminate the OCC stripes in images. The band-pass filter converts the OCC noised image from the time domain to the frequency domain via fast Fourier transform, then uses a filter to remove the features related to OCC stripes. To quickly verify the OCC noised image restoration performance, we show a visualization comparison between the band-pass filter method and the IR module, as shown in Figure 6. We can find that the band-pass filter method results in distortions in some pixel spaces and brings gray stripes, while the IR module basically eliminates the occlusion of stripes, leaving a clean license plate. In particular, the peak signal-noise-ratio (PSNR) scores of the recovered images by our IR module are almost over 30 dB, obtaining an improvement of about 8.84 dB over the band-pass filter method, which can preliminarily demonstrate the effectiveness of the proposed scheme in terms of mitigating OCC’s negative effect on license-plate recognition, we report the performance of the proposed license-plate recognition scheme in detail following.

### 4.1. License-Plate Detection (LPD) Accuracy

**Figure 7:**
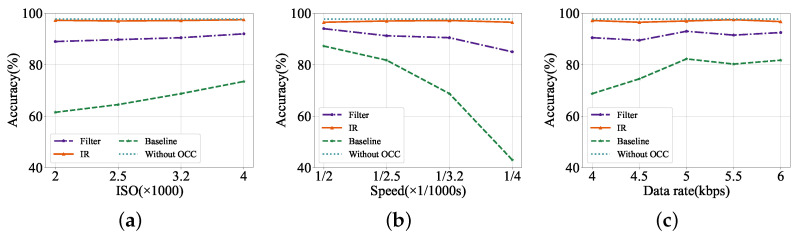
Detection accuracy under varying settings on the synthesized dataset. (**a**) Increasing ISO. (**b**) Increasing shutter speed. (**c**) Increasing data rate.

As locating the license-plate region in received frames is essential for recognizing characters, we first report the license-plate detection accuracy. In our experiments, the bounding box of a license plate is considered to be detected when its IoU with the ground truth bounding box is more than 70%. We mainly explore how the camera’s ISO, shutter speed, and transmission data rate impact license-plate detection performance, and results are shown in Figure 7. Generally, the average license-plate detection accuracy is close to 97.75% without OCC stripes interference under all settings. However, the OCC stripe pattern has a negative effect on license-plate detection accuracy; hence, the baseline’s performance is dramatically degraded, and the average detection accuracy has a maximum 54.75% drop, while the proposed scheme has only slight fluctuation in accuracy. Figure 7a further shows the results under increasing ISO with a fixed shutter speed of 1/3200 s and data rate at 4 kbps. We can find that the detection accuracy of the image reconstruction module is 97.23% on average, which is 6.92% higher than the accuracy of the band-pass filter method. Figure 7b reports the results under varying shutter speeds with fixed ISO of 3200 and 4 kbps transmission rate. The average accuracy of the baseline is 70.18%, and the band-pass filter method improves the accuracy to 90.20% while the IR module brings an improvement to 96.82%.

Additionally, we can find that the varying ISO and shutter speed settings barely affect the detection accuracies after the image reconstruction module. The results prove that the image reconstruction module is suitable for scenes with different camera parameter settings. We then explore the constructive effect of the band-pass filter method and IR module under varying data rates with fixed 3200 ISO and 1/3200 s shutter speed as shown in Figure 7c. It can be seen that the average detection accuracy of the band-pass filter method is 91.4%, while the IR module is 96.98%. It should be noted that a low data rate more likely leads to poor detection accuracy, as shown in the baseline’s result under 4 kbps. It can be explained that a lower data rate means wider OCC stripes in captured images, hence increasing the difficulty of image restoration, while the image reconstruction module maintains high detection accuracies under varying data rates. The above results verify that our image reconstruction module can effectively improve the license-plate detection accuracy under the interference of the OCC stripes in the OCC-based vehicle networks, and has a better performance than the band-pass filter method.

### 4.2. License-Plate Recognition (LPR) Accuracy

In this subsection, we evaluate the LPR accuracy under varying experiment settings with synthesized data and real-scene data.

#### 4.2.1. LPR Accuracy on the Synthesized Dataset

**Figure 8:**
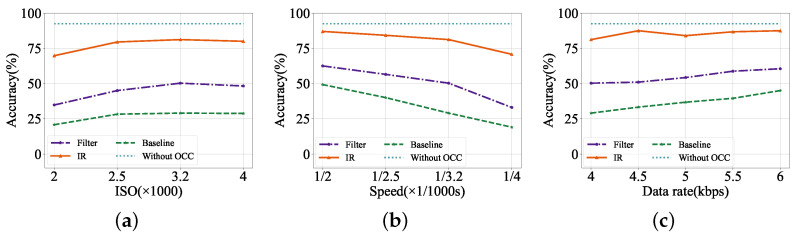
Recognition accuracy with different settings on the synthesized dataset. (**a**) Increasing ISO. (**b**) Increasing shutter speed. (**c**) Increasing data rate.

We first evaluate the license-plate recognition accuracy of the proposed scheme on the synthesized dataset. Specifically, we explore the impact of various ISO, shutter speed, and transmission data rates on recognition accuracy, and results are shown in Figure 8. The average LPR accuracy under various experimental settings is about 92.50% without OCC interference. However, the recognition accuracy of the baseline is only 32.88% on average, proving that the OCC stripes have a significant negative impact on the LPR accuracy in the OCC-based vehicle networks.

Figure 8a shows the recognition accuracy of the band-pass filter method and our IR module under increasing ISO with a fixed shutter speed of 1/3200 s and 4 kbps data rate. We find that the average recognition accuracy of our IR module is 77.61% on average, which is 33.05% higher than the band-pass filter method. Moreover, we also study the gain of the band-pass filter method and IR module to bring to the recognition accuracy under varying shutter speeds where the ISO is fixed to 3200, and the data rate is fixed to 4 kbps. As is shown in Figure 8b, the band-pass filter method makes the accuracy increase from 34.31% to 50.56% on average, while the recognition of our IR module is 30.24% higher than the band-pass filter method. The recognition accuracy of both the band-pass filter method and IR module tends to rise with the ISO increasing and the shutter speed slower. It can be explained that a higher ISO and slower shutter speed mean more received light intensity, thus promoting the salience of license-plate features. Furthermore, we explore the positive effect of the two methods under different data rates with a fixed ISO of 3200 and a shutter speed of 1/3200 s. It can be seen in Figure 8c that the average recognition accuracy of the band-pass filter method and IR module are 54.95% and 85.39%, respectively. We find that the accuracy of the baseline slightly increases as the data rate increases, and by contrast, the IR module is more insensitive to varying data rates. The recognition accuracy of the IR module hardly changes much, no matter how the data rate varies. The result above demonstrates that the image reconstruction module is more robust than the band-pass filter method, hence having the potential to be applied in real scenes of the OCC-based vehicle networks.

#### 4.2.2. LPR Accuracy in Real Scenes

**Figure 9:**
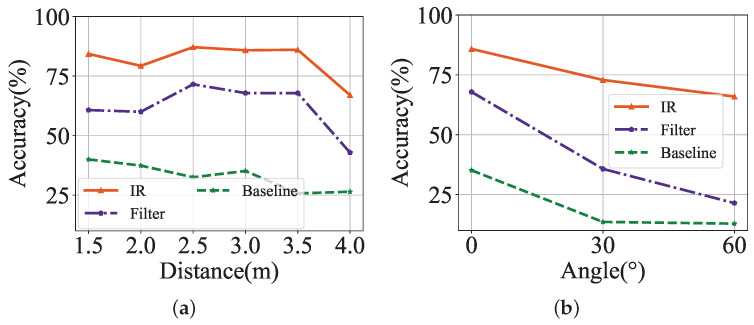
Recognition accuracy under varying experiments in real scenes of the OCC-based vehicle networks. (**a**) Increasing distance. (**b**) Varying angle.

We further evaluate the proposed license-plate recognition scheme in real scenes of the OCC-based vehicle networks. Considering the IR module’s recognition accuracy is relatively insensitive to varying ISO, shutter speed, and data rates as studied in the synthesized dataset, we fixed them as 3200, 1/3200 s, and 5 kbps, respectively. We mainly explore the impact of distance and angle between the license plate and receiver to mimic the realistic scenario, and results are shown in Figure 9. As illustrated in Figure 9a, the average recognition accuracy of the IR module is 81.60% under varying distances, significantly exceeding the band-pass filter’s 61.79% and the baseline’s 32.86%. Furthermore, we find that the results of all methods have a slight drop in relatively close recognition distances. It can be explained that close distances lead to light saturation in captured images, thus degrading the recognition accuracy, this problem can be readily solved by setting automatic adjustment of camera ISO. Meanwhile, longer distance makes it more difficult to recognize the area of the license plate. This problem can be addressed by adding a lens to the LED or increasing the LED power. These adjustments can increase the recognition distance of the system to over ten meters in real scenes of the OCC-based vehicle networks. In our experimental scenario, we finally conclude that the 3 m is probably the most optimal recognition distance and fix the distance for the following experiment. We then explore the recognition accuracy under varying angles between the license plate and receiver, and the results are shown in Figure 9b. We find that a larger angle increases the difficulty of recognition, thus reducing the accuracy. For example, in our experiments, we find that the system incorrectly recognizes the character ‘D’ as ‘0’ when the angle is set to $60^{{}^{\circ}}$ like a human-made error.

On the whole, the proposed image reconstruction module can achieve a recognition performance of 79.35% in real scenes of the OCC-based vehicle networks with various settings of the distances and angles between the license plate and receiver, which is 24.26% higher than the band-pass filter method. It confirms the feasibility of our scheme in real-scene OCC-based vehicle networks.

### 4.3. LPR Accuracy Comparisons

In this subsection, we conduct comparison experiments to evaluate the performance of the proposed LPR system. First, we evaluate the NAFNet module in license-plate IR. For a fair comparison, we fix the shutter speed, ISO, and data rate with 1/3200 s, 3200, and 4 kbps, respectively, compared to additional IR methods in synthesized and real-scene data [31,32,33]. From Table 1, we can find that the NAFNet-based IR module obtains higher LPR accuracy than other methods, which demonstrates the effectiveness of our proposed method. Sequentially, we adopt the proposed IR module to compare with baseline methods in these two sorts of dataset, under the 1/3200 s shutter speed, 3200 ISO, 5 kbps data rate, 3 m distance and $0^{\circ }$ angle, the results are shown in Table 2. We can observe synthesized data obtain higher accuracy across different methods. This is attributed to the fact that synthesized OCC corrupted license-plate images avoid interference of ambient noise.

## 5. Conclusions

To deal with the problem that existing LPR systems in potential applications of the IoV may be affected by OCC, we propose a pipeline scheme with an image reconstruction module to repair the pixels damaged by modulated light in captured images in the vehicle networks and minimize the impact of OCC stripes on license-plate frames. We separately make a synthesized dataset and capture realistic license-plate frames with OCC stripes via a real-scene prototype to evaluate the performance of our scheme. Compared with the band-pass filter method, the experimental results show the proposed image reconstruction module can improve the detection accuracy by over 6.31% on the synthesized dataset. The recognition accuracy is improved by more than 31.18% on average on the synthesized dataset and 24.26% in real scenes, which strongly demonstrates the effectiveness of the proposed scheme. Moreover, our image reconstruction takes up less than 110 Mb of storage space and has the potential to be embedded into existing license-plate recognition systems and other applications in the IoV. We are actively seeking relevant scenarios in the OCC-based vehicle networks for deploying the proposed scheme to promote the development of intelligent traffic.

## Figures and Tables

**Table 1:** Accuracy comparison of different IR methods on synthesized and real-scene data.

Methods	Synthesized Data	Real Scenes Data
NAFNet	83.14%	79.43%
Noise2void [33]	62.85%	64.79%
AP-BSN [31]	75.29%	67.72%
DBSN [32]	69.16%	58.36%

**Table 2:** Comparison of accuracy between synthesized data and real-scene data.

	IR	Filter	Baseline
Synthesized data	86.52%	64.31%	39.26%
Real scenes data	79.37%	62.19%	36.83%

## Data Availability

The original contributions presented in the study are included in the article, further inquiries can be directed to the corresponding author/s.
